# Development of a Machine Learning Interatomic Potential for Zirconium and Its Verification in Molecular Dynamics

**DOI:** 10.3390/nano15211611

**Published:** 2025-10-22

**Authors:** Yuxuan Wan, Xuan Zhang, Liang Zhang

**Affiliations:** 1 International Joint Laboratory for Light Alloys (MOE), College of Materials Science and Engineering, Chongqing University, Chongqing 400044, China; 2 State Key Laboratory of Mechanical Transmission for Advanced Equipment, Chongqing University, Chongqing 400044, China

**Keywords:** deep potential, first-principles calculations, molecular dynamics, machine learning, zirconium

## Abstract

Molecular dynamics (MD) can dynamically reveal the structural evolution and mechanical response of Zirconium (Zr) at the atomic scale under complex service conditions such as high temperature, stress, and irradiation. However, traditional empirical potentials are limited by their fixed function forms and parameters, making it difficult to accurately describe the multi-body interactions of Zr under conditions such as multi-phase structures and strong nonlinear deformation, thereby limiting the accuracy and generalization ability of simulation results. This paper combines high-throughput first-principles calculations (DFT) with the machine learning method to develop the Deep Potential (DP) for Zr. The developed DP of Zr was verified by performing molecular dynamic simulations on lattice constants, surface energies, grain boundary energies, melting point, elastic constants, and tensile responses. The results show that the DP model achieves high consistency with DFT in predicting multiple key physical properties, such as lattice constants and melting point. Also, it can accurately capture atomic migration, local structural evolution, and crystal structural transformations of Zr under thermal excitation. In addition, the DP model can accurately capture plastic deformation and stress softening behavior in Zr under large strains, reproducing the characteristics of yielding and structural rearrangement during tensile loading, as well as the stress-induced phase transition of Zr from HCP to FCC, demonstrating its strong physical fidelity and numerical stability.

## 1. Introduction

Zirconium (Zr) is widely used in the manufacture of key components for nuclear reactors due to its low neutron absorption rate, excellent corrosion resistance, and high-temperature stability, such as fuel cladding and pressure tubes [1]. In the complex operating environment of nuclear reactors, the performance evolution of Zr and its alloys is influenced by the combined effects of various factors, including neutron irradiation and high temperatures. Neutron irradiation can cause point defects such as vacancies and interstitial atoms to form within the material, and may induce microstructural changes such as dislocation loops and void swelling, thereby affecting the mechanical properties and dimensional stability of materials [2,3,4]. High temperatures accelerate the diffusion process of atoms, leading to creep and phase transformation, which in turn affects the long-term service performance of materials [5,6,7,8]. In addition, the corrosion behavior of Zr alloys in high-temperature steam environments can lead to the formation of oxide layers, and the growth and cracking of these oxide layers may further exacerbate the degradation of material performance [9]. These factors work together to determine the performance evolution patterns and failure mechanisms of Zr alloys in nuclear reactors. Experimental studies have played an important role in gaining a deeper understanding of how these factors affect the properties of Zr and its alloys [10,11], but they are costly, time-consuming, and difficult to precisely control and observe phase transformations, defect motion, and oxide layer growth processes at the atomic scale. Computational simulation is an important tool for studying the properties of Zr and its alloys. Theoretical models and numerical methods can simulate and analyze how structural evolution, such as phase transitions and defect formation, relates to macroscopic properties [12,13,14,15,16,17], predict the mechanical properties, deformation characteristics, and corrosion resistance of Zr alloys under different environments, and provide theoretical support for material design and experimental research.

Atomic-scale computational simulations mainly include the first-principles calculations based on density functional theory (DFT) and molecular dynamics (MD) simulations. However, the high computational cost of DFT limits its application in large-scale systems [18]. MD simulations can handle systems containing millions or even hundreds of millions of atoms and has high computational efficiency [19,20], but its accuracy is limited by the accuracy of the potential [21]. Traditional interatomic potentials are derived from empirical methods and often lack the accuracy and transferability required for complex systems [22,23,24]. At normal temperature and pressure, metallic Zr primarily exists in the hexagonal close-packed (HCP) structure, which has relatively low symmetry. This results in more complex atomic arrangements and bonding characteristics compared to face-centered cubic (FCC) or body-centered cubic (BCC) structures. Under irradiation conditions, point defects such as vacancies and interstitial atoms form in Zr. The migration energy and binding energy of these defects are closely related to the local environment of the surrounding atoms. Empirical potentials often fail to accurately describe the interatomic interactions in such complex local environments [25]. Moreover, when studying the phase transition behavior of Zr, the transformation from HCP to FCC involves significant changes in lattice symmetry, while empirical potentials lack the accuracy to describe the energy barriers and transition states associated with such phase transitions [26]. These limitations restrict the application of empirical potentials in simulating the complex phase transition dynamics of Zr.

In recent years, machine learning interatomic potentials trained on first-principles data have overcome the above limitations and gradually become a research hotspot in the field of materials simulation [24]. By using deep neural networks to fit data from DFT, these potentials can significantly improve both accuracy and applicability, enabling large-scale simulations while maintaining the computational accuracy of DFT. As one of the most widely used machine learning algorithms, neural networks can learn complex interatomic interactions from large datasets of atomic structures, thereby achieving higher accuracy in multi-atom systems and complex chemical environments [27,28,29,30]. With the increasing complexity of neural network frameworks, deep learning has been proposed and rapidly applied across various fields. As a key branch of deep learning, Deep Potential (DP) has become a core technology in the development of neural network potentials, owing to its powerful modeling capabilities and efficient data processing [31,32,33]. Today, the DP method has made significant progress in the structural prediction and dynamic behavior studies of metallic and alloy systems, including single-element metals such as Li [34,35], Sc [36], and W [37], as well as complex multicomponent alloys such as Al-Mg [38], Ag-Si [39], Mg-Al-Si [40], and Al-Cu-Mg [41].

Therefore, this study aims to develop a high-precision machine learning potential for Zr using a “DFT + DP + MD” method. This paper first describes the dataset construction process, including crystal structure sampling, defect state generation, and thermal disturbance dynamic extraction, to ensure that the training data has good representativeness and diversity in the configuration space. Second, model training is conducted under the DP-GEN framework, combining data screening, model iteration, and error monitoring strategies to construct a physically consistent neural network potential. To validate the reliability of model, quantitative assessments are conducted across multiple dimensions, including fundamental properties (lattice constants, surface energy, grain boundary energy), thermodynamic stability (thermal expansion coefficient, melting point), and mechanical properties (elastic modulus, bulk modulus, stress–strain response). These results are then compared in detail with DFT, typical empirical potential results, and experimental data. Finally, the advantages of this neural network potential in simulating complex structural evolution processes, thermodynamic behavior, and mechanical responses are summarized, and its potential applications in high-throughput material screening and multiscale modeling in future research are explored.

## 2. Materials and Methods

### 2.1. Dataset Construction

**Figure 1:**
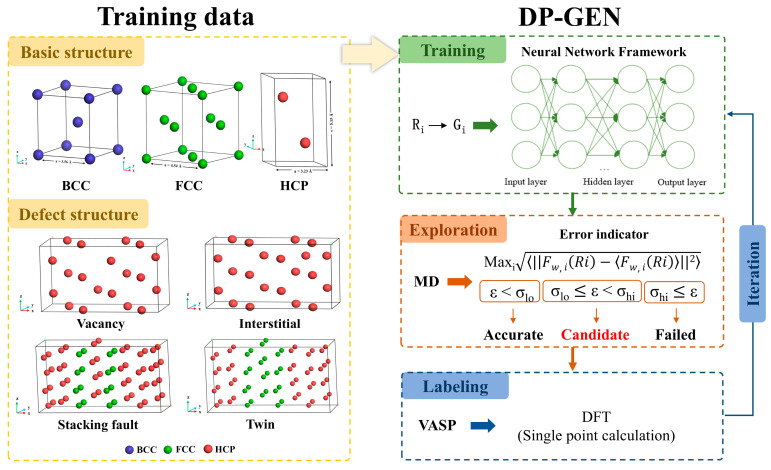
Crystal structure information in the initial dataset and flowchart of neural network potential training in DP-GEN framework [48].

High-quality initial datasets need to cover information about Zr in different structural and physical states to ensure that the trained potential can accurately predict material properties under a wide range of conditions. The initial dataset used in this study consisted of two parts. The first part includes three fundamental structures of Zr: HCP, FCC, and BCC. Unit cells for each structure were initially created using VESTA [42] and then expanded. The HCP and BCC structures contain 64 atoms, while the FCC structure contains 72 atoms. The second part consists of Zr defect structures, including vacancy, interstitial, twin, and stacking fault configurations, as illustrated in Figure 1. Aiming to cover structures under different strains, we scaled the lattice constants of the bulk structures by ±10%. To ensure the reasonable structures of both the fundamental and defect configurations, structural optimization was first performed. Subsequently, to obtain nonequilibrium zirconium structures of Zr at different temperatures, ab initio molecular dynamics (AIMD) simulations were conducted at four temperatures (300 K, 800 K, 1300 K, and 1800 K), with 1000 simulation steps at each temperature and one structure sampled every 5 steps. Finally, high-precision single-point energy calculations were performed on the sampled structures from each temperature. Both the AIMD simulations and single-point energy calculations are performed using the Vienna Ab initio Simulation Package (VASP) [43] based on first-principles methods. The calculations employ a plane-wave pseudopotential approach within the framework of DFT, using the Perdew-Burke-Ernzerhof (PBE) exchange-correlation functional under the generalized gradient approximation (GGA) to describe electron exchange and correlation effects [44]. For AIMD calculations, we chose single Gamma points to improve computational efficiency. To ensure the accuracy of the single-point energy calculations, convergence tests were conducted for the cutoff energy and k-point mesh. As a result, the plane-wave cutoff energy was set to 400 eV, the energy convergence criterion was set to 1 × 10^−6^ eV, and the k-point density was 0.025 Å^−1^. Brillouin zone sampling was performed using the Monkhorst-Pack scheme [45]. There are 2400 data samples were generated for the three fundamental structures. Each data sample contains atomic configurations along with their corresponding positions, energies, interatomic forces, and stress information. The final dataset comprises 4049 samples in total, which are split into training and validation sets at a ratio of 3:1. The training set is used to fit the neural network potential, while the validation set is employed to assess the generalization ability and predictive accuracy of model.

### 2.2. DP-GEN Model

The Deep Potential Generator (DP-GEN) framework [46,47] is used to develop a neural network potential for Zr. DP-GEN is an efficient potential generation method based on Active Learning and DFT, and its core idea is to continuously expand the training set through multi-stage iteration, to gradually improve the accuracy and applicability of the potential while efficiently exploring the material configuration space, and ultimately construct the high-quality DP models. Its main workflow includes three steps of training, exploration, and labeling, as shown in Figure 1.

The training stage is the first step in the DP-GEN workflow, aiming to train the DP model based on the available training dataset. In this study, the descriptor type is set to se_e2_a, which transforms interatomic distances R_i_ into feature matrices G_i_ through an embedding network. The final output is a feature vector that describes the local atomic environment, suitable for capturing many-body interactions. The cutoff radius is set to 7.0 Å, with a smoothing radius of 0.5 Å to ensure a smooth transition near the boundary. The neural network structure of the descriptor consists of three layers with the number of neurons in each layer being 20, 40, and 80, respectively, and this progressively increasing structure helps to extract more complex features. The fitting network adopts a structure of 240 neurons in each of the three layers and introduces a residual network to improve training efficiency and model stability. The initial learning rate is 0.001, and an exponential decay strategy is used to decay every 5000 steps. The training part sets the total number of training steps to 350,000, and four different DP models are generated for each training, and the only difference between these models is the random seed for the initialization of the neural network.

After the training is completed, then move to the exploration stage. This stage involves discovering new atomic configurations by implementing DPMD simulations in combination with MD and DP to expand the training set and improve the accuracy and generalization of the model. The explored structures selected in this stage include the perturbed structures of HCP, FCC, and BCC, whose lattice constants are all adjusted according to the deflation coefficients ranging from 0.9 to 1.1 with 0.05 intervals to cover the configuration space in different compression and tension states, in addition to the hexagonal ω substable-phase structure and the twin-crystal structure. All the explored structures were explored using the four DP models generated in the training phase, and MD simulations were carried out at seven temperatures, 300, 600, 900, 1200, 1500, 1800, and 2100 K, and three pressures, 0.1, 0.5, and 1 GPa, under the NVT or NPT system, with a total of 18 iterations of exploration. In addition, model deviation trust was set as a criterion for screening candidate configurations during the exploration process, and the formula is shown in Figure 1, with an upper limit of σ_hi_ = 0.35 and a lower limit of σ_lo_ = 0.10. The model deviation ε was calculated for each explored configuration. When ε < σ_lo_, it indicates that the DP model can accurately predict the configuration. When σ_lo_ < ε < σ_hi_, the explored configuration was selected as a candidate configuration, and ε > σ_hi_ means that the prediction uncertainty of the configuration is too high, and for computational stability and resource control reasons, such configuration cannot go to the next stage.

After exploration, then move to the labeling stage to perform single-point energy calculations on the candidate configurations selected in the exploration step and add them to the training set for the next round of iterative training. In this study, the upper limit of the number of candidate configurations to be calculated is set to 50. If the number of candidate configurations is more than 50, 50 of the structures are randomly selected for single-point calculation. If the number of candidate configurations is less than 50, all the structures are subjected to single-point calculation. All calculations are performed using VASP with the same parameter settings as the initial training set of single-point energy calculations.

After the completion of the above three stages, the next round of training stage is carried out to improve the prediction accuracy of the DP model through continuous iterations. In each iteration, the high-precision data computed in the labeling stage are merged into the initial training set, and the updated and more comprehensive training set is used as the input for the next iteration to restart the training step. Specific parameters such as the maximum number of iterations specified during the iteration process, the number of configurations explored in each iteration, and the energy or force thresholds for screening configurations are set in the input file. The maximum number of iterations specified in this study is 18, and four fully optimized DP models are output after the iterations are completed.

## 3. Results

### 3.1. DP Training and Accuracy Evaluation

**Figure 2:**
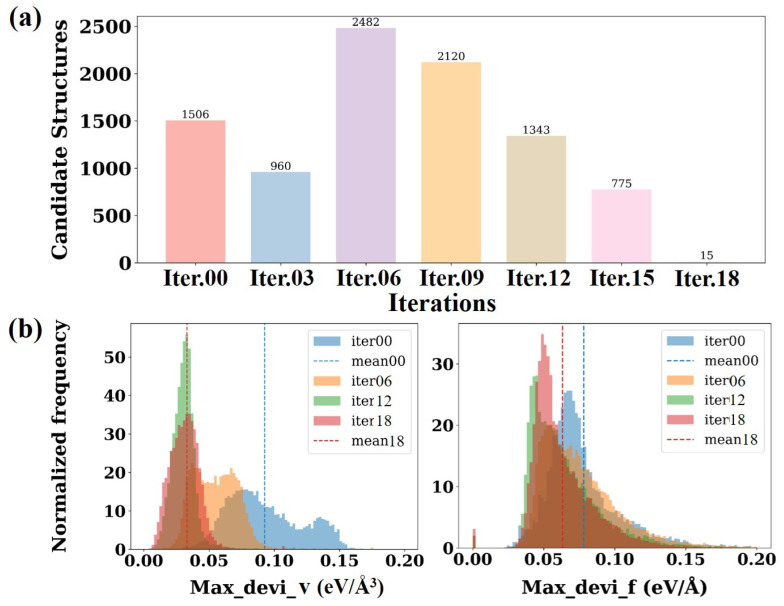
(**a**) Statistics of the number of candidate configurations, (**b**) Statistics of the maximum deviation in stress tensor/interatomic force predictions during the DP-GEN iterative process.

Figure 2a illustrates the trend of the number of candidate conformations generated during DP-GEN iterations and the DP model prediction error with iteration rounds. The number of candidate conformations generated at the beginning of the iteration (iter.00 to iter.06) is relatively high, with iter.02 reaching the maximum value of 2482 conformations. Thereafter, as the iteration proceeds (iter.09 to iter.18), the number of candidate configurations gradually decreases and reaches a minimum value of 15 at iter.18, indicating that with the continuous optimization of the model, the ability to describe the target system gradually improves, and it can be assumed that the current training set has basically contained the main physical features required. Figure 2b presents a statistical analysis of the maximum force deviation (Max_devi_f) and maximum stress tensor deviation (Max_devi_v) of the candidate configurations at each iteration stage. In DP-GEN, Max_devi_f and Max_devi_v represent the standard deviations of the predicted atomic forces f_i_ and stress tensors $\Xi_{\alpha \beta }$, respectively, obtained from the four DP models. These metrics are commonly used as key indicators for evaluating the prediction accuracy of a given structure and are defined as follows [49,50].

(1) $$\mathrm{Max\_devi\_f}=\sqrt{\langle {\left|\right|f_{i}-\bar{f_{i}}\left|\right|}^{2}\rangle },\;\bar{f_{i}}=\langle f_{I}\rangle$$

(2) $$\mathrm{Max\_devi\_v}=\;\frac{1}{N}\sqrt{\langle {\left(\Xi_{\alpha \beta }-\;\bar{\Xi_{\alpha \beta }}\right)}^{2}\rangle },\;\overset{-}{\Xi_{\alpha \beta }\;}=\langle \Xi_{\alpha \beta }\rangle$$

In Equation (1), i denotes the atomic index, iterating over all atoms in the structure; f_i_ represents the atomic force vector predicted by a specific DP model for atom i, and $\bar{f_{i}}$ is the average force predicted by the four DP models for that atom. In Equation (2), $\Xi_{\alpha \beta }$ refers to the stress component in the direction combination of α and β, while $\bar{\Xi_{\alpha \beta }}$ denotes the average stress predicted by the four DP models. N represents the number of atoms in the system, and $\langle \cdot \rangle$ indicates the average over the model ensemble. The distribution of maximum force deviations reveals that, as the number of iterations increases, the frequency of force deviations shifts progressively toward lower deviation intervals, and the average values for each iteration (indicated by dashed lines) also decrease accordingly. This indicates that the accuracy of predicted interatomic forces improves with successive iterations. A similar trend is observed in the distribution of maximum stress tensor deviations. As iterations proceed, the maximum stress deviations associated with different configurations become lower and more concentrated, with their average values gradually decreasing. The result suggests that the predictions of stress responses across configurations by the model are becoming increasingly accurate, and its overall stability and reliability are continually improving.

**Figure 3:**
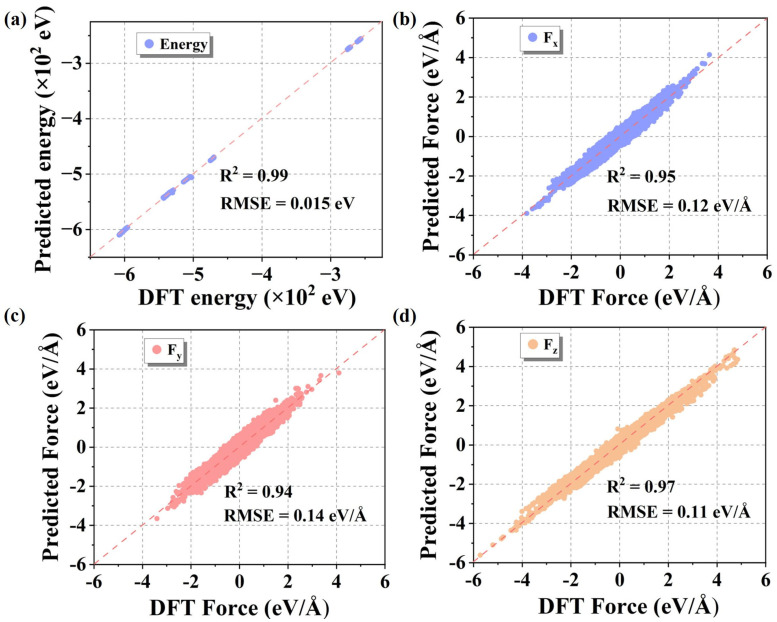
Comparison between the results predicted by DP and those calculated by DFT. (**a**) Total energy of the structures, (**b**) atomic forces in the x-direction, (**c**) atomic forces in the y-direction, and (**d**) atomic forces in the z-direction.

The trained DP models was applied to the structures in the validation set to calculate the total energy and atomic forces in each direction. These predictions were then compared with the corresponding DFT results, as shown in Figure 3. Figure 3a presents a scatter plot of the predicted total energy versus the DFT energy, where the data points are highly concentrated near the diagonal line, indicating strong consistency. Figure 3b–d show the predicted force components in the x, y, and z directions compared with DFT-calculated values, also demonstrating strong linear correlations. To quantitatively assess model performance, error statistics were conducted for all validation data. The results show that the root mean square error (Energy RMSE/Natoms) for energy predictions is 2.86 meV/atom, and the root mean square error (Force RMSE) for atomic forces is 94 meV/Å, indicating excellent prediction accuracy. These findings demonstrate that the DP models achieve high precision in predicting both energy and force, two key physical quantities.

### 3.2. Verification of Basic Properties

Basic structural properties of metals such as lattice constants, surface energy and grain boundary energy are important benchmarks in the validation of machine learning potentials. Lattice constant reflects the accuracy of the model’s description of the equilibrium crystal structure. Surface energy tests the performance and stability of the potential in dealing with localized atomic arrangement disorder and coordination defects. Grain boundary energy examines its ability to generalize to complex asymmetric localized environments. These physical quantities cover the multi-scale features involved from the ideal crystal structure at the atomic scale to higher-order defect structures, and can effectively evaluate the ability of the potential to model different atomic environments. By systematically evaluating these key physical quantities, the applicability and stability of the DP in different structural systems can be examined.

#### 3.2.1. Lattice Constant

**Figure 4:**
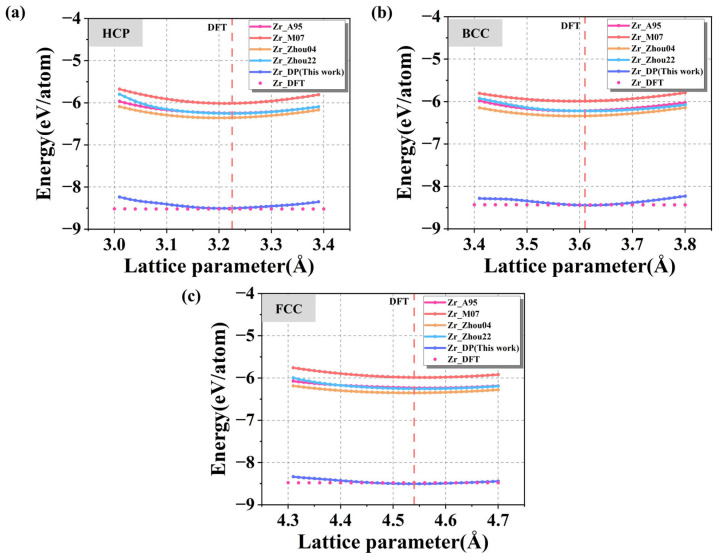
(**a**) HCP-Zr; (**b**) FCC-Zr; (**c**) BCC-Zr Cohesive energy versus lattice constant curves under different potential functions and DFT calculations.

Figure 4a–c show the energy-lattice constant relationships for HCP, FCC, and BCC crystal structures predicted by four commonly used empirical potentials (Zr_A95 [50], Zr_M07 [25], Zr_Zhou04 [51], Zr_Zhou22 [52]) and the DP model, with DFT results used as the reference. As shown in the figures, the energies corresponding to the equilibrium lattice constants calculated by DP in the three crystal structures are the closest to the results of the DFT calculations, while the deviations of the energies calculated by using other potential functions from the DFT benchmark results are generally more than 2 eV/atom, which is relatively high, indicating that the traditional potentials are not sufficiently accurate in describing crystal energies, and it is difficult to accurately capture the fine relationship between energies and the lattice variations on the atomic scale. The subtle relationship between energy and lattice changes at the atomic scale is difficult to be accurately captured. A more detailed comparison of the Zr equilibrium lattice constants predicted by various potentials with DFT for the three structures, along with the surface and grain boundary energy results discussed later, is shown in Table 1. It can be seen that DP accurately achieves the accuracy of DFT in all the three structures, with a = 3.22 Å and c = 5.14 Å in the HCP structure, which are almost no different from the DFT results (3.23 Å and 5.15 Å), and high agreement is also achieved for both the BCC and FCC structures. In contrast, the traditional potentials has a deviation of 0.01~0.03 Å in some structures, indicating that the DP describes the crystal structure more accurately and possesses a better structural generalization ability.

#### 3.2.2. Surface Energy

**Figure 5:**
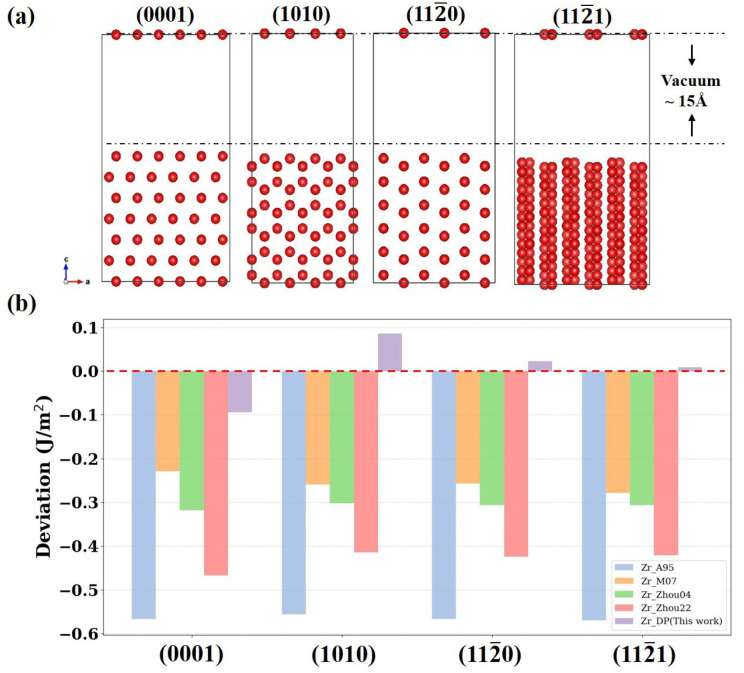
(**a**) Atomic structure diagrams of the (0001)\(1010)\(11$\overset{-}{2}$0\(11$\overset{-}{2}$1) surfaces of Zr. (**b**) Analysis of the deviation between the surface energies calculated by different potentials and the DFT results. The vertical coordinates indicate the deviation between the calculated values of each potential function and the DFT results, with negative values representing underestimation and positive values representing overestimation.

Surfaces represent typical non-equilibrium structures in materials, where atomic bonding states differ significantly from those inside an ideal crystal. Figure 5a shows schematic atomic structures of four surfaces (0001), (1010), (11$\overset{-}{2}$0), and (11$\overset{-}{2}$1) of α-Zr. Each model adopts a slab configuration with a 15 Å vacuum layer above the surface to accurately simulate a free surface environment. Figure 5b demonstrates the deviation of the surface energy results calculated by four empirical potentials (Zr_A95, Zr_M07, Zr_Zhou04, Zr_Zhou22) and DP relative to the DFT calculations. The results show that the traditional empirical potentials are significantly underestimated on all crystal surfaces, with the deviation of the EAM potential Zr_A95 as high as about 0.6 J/m^2^. This suggests that the empirical potentials have limited physical description and generalization capabilities when dealing with structures with low symmetry. In contrast, the surface energy predictions of DP on all crystal planes are highly close to the DFT results, with deviations within ±0.1 J/m^2^, showing good anisotropy resolution. Moreover, these surface structures are not included in the training data of DP, so this result further validates the ability of the model in capturing complex interatomic interactions in a low-symmetry environment involving surface effects in response to non-training structures.

#### 3.2.3. Defect Energetics

Defect migration energy is an important physical quantity that characterizes the mobility of point defects (such as vacancies and interstitial atoms) in materials, and it has a decisive influence on diffusion behavior, plastic deformation, phase transformation kinetics, and irradiation damage evolution. Therefore, this section compares the migration energies of typical point defects in HCP-Zr, including vacancy migration energy and interstitial atom migration energy. Specifically, we employed four empirical potentials and DP to calculate the energy barriers for single vacancy migration along the basal plane and *c*-axis, as well as the migration energy barriers for interstitial atoms within the basal plane using the LAMMPS-NEB method. These results were compared with DFT calculations to evaluate the descriptive accuracy of DP across different diffusion pathways. The results are shown in Table 1. The vacancy basal plane migration energy predicted by DP is approximately 0.757 eV, while the migration energy along the *c*-axis is about 0.823 eV. The migration energy barrier for interstitial atoms within the basal plane is approximately 0.632 eV. These values are slightly higher than the DFT results (0.54, 0.65, and 0.27 eV, respectively). In contrast, other empirical potentials exhibit significantly greater deviations in predicting defect migration barriers, reaching as high as 3 to 4 eV. This indicates that despite some overestimation of DP in the high-energy transition state region, they can still reasonably reflect the intrinsic characteristics of point defect migration dynamics in HCP-Zr. In contrast, traditional empirical potentials, due to their rigid potential energy surfaces and lack of adaptive description for local environment changes, often exhibit significant deviations in high-energy states or highly distorted structures, making it difficult to accurately characterize the actual diffusion process.

**Figure 6:**
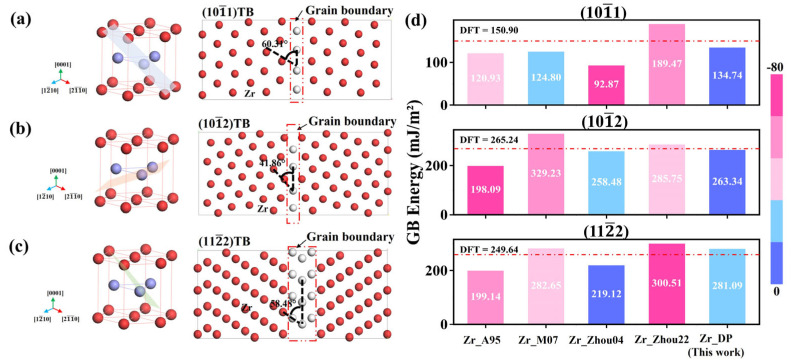
Twin boundaries of Zr (**a**) (10$\overset{-}{1}$1), (**b**) (10$\overset{-}{1}$2), and (**c**) (11$\overset{-}{2}$2). (**d**) Boundary energies of the three twin boundaries and their errors with respect to the DFT calculations computed with different traditional potentials. The red dashed line is the result of the DFT calculations, and the color of the columns from red to blue indicates that the error is from the largest to the smallest.

To further evaluate the applicability of the DP in more complex defect systems, this section examines its ability to describe grain boundary structures and energies. Compared to the ideal crystal structure, grain boundaries are usually accompanied by lattice distortions, coordination number changes, and breakage of long-range ordering. In order to test the accuracy of DP in describing the grain boundary energies, three typical twin boundaries of Zr, (10$\overset{-}{1}$1), (10$\overset{-}{1}$2), and (11$\overset{-}{2}$2) are selected. As shown in Figure 6a–c, the left side shows the single-cell crystal orientation and the right side shows the atomic arrangement of the constructed slab structure along the cross-section direction of the grain boundary. Figure 6d shows the grain boundary energies predicted by different potentials for the three twin grain boundaries of Zr and compares them with the results of DFT calculations. From the data, the prediction accuracies of the traditional potentials on different structures vary widely, with general underestimation or overestimation and limited ability to discriminate the energies of different grain boundary types. For example, the prediction of Zr_A95 on the (10$\overset{-}{1}$1) is only 120.93 mJ/m^2^, which is significantly lower than that of 150.90 mJ/m^2^ from DFT calculations, showing a clear tendency of underestimation, and the prediction of Zr_Zhou04 for this grain boundary is even lower at 92.87 mJ/m^2^, with the largest error. For the (10$\overset{-}{1}$2) and (11$\overset{-}{2}$2) grain boundaries, Zr_M07 and Zr_Zhou22 exhibit relatively higher predicted values, but still have significant deviations from the DFT results. In contrast, the predictions of DP on the three grain boundaries are 134.74, 263.34, and 281.09 mJ/m^2^, respectively, all of which are closer to the DFT results (150.90, 265.24, and 249.64 mJ/m^2^). In particular, the prediction on the (10$\overset{-}{1}$2) grain boundary is only 1.9 mJ/m^2^ lower than DFT, showing good generalization performance and sensitive response ability to the structural energy change of the grain boundary. In addition, for the (11$\overset{-}{2}$2), although the DP prediction value is slightly higher than the DFT result, its deviation is still much smaller than that of the traditional potentials, and this difference can be regarded as a reasonable error, which further corroborates the generalization performance and reliability of the DP for the modeling of grain boundaries.

### 3.3. Verification of Thermodynamic and Mechanical Properties

Mechanical properties such as elastic constant (C_ij_), bulk modulus (B_V_), shear modulus (G_V_) and tensile strength are the core parameters that characterize the structural response of a material against external mechanical loads (e.g., stresses and strains), and they directly reflect the rigidity, anisotropy, and plastic deformation behaviors of the crystal structure. The thermodynamic properties, such as thermal expansion coefficient and melting point, are closely related to the phase stability, atomic vibrational behavior, and energy barriers of the materials under different thermodynamic conditions, and are important indicators for describing the thermal stability, phase transition paths, and atomic-scale structural reconstruction process of the materials. By calculating and analyzing these key mechanical and thermodynamic properties and quantitatively comparing them with experimental data and DFT, the predictive ability and physical consistency of DP in different properties of Zr can be assessed.

#### 3.3.1. Thermal Expansion and Melting

**Figure 7:**
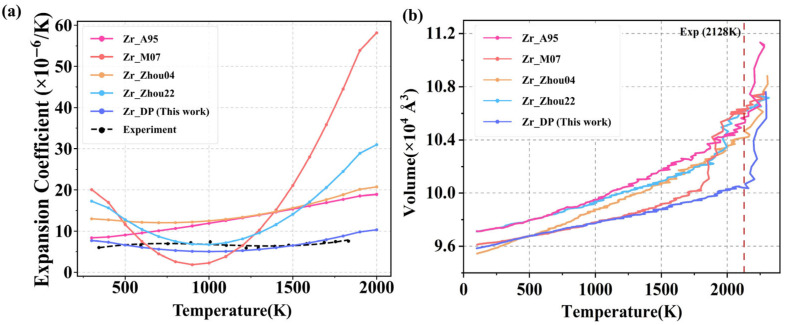
(**a**) The temperature dependence of thermal expansion coefficients predicted by different potentials and measured experimentally [58]. (**b**) HCP-Zr volume versus temperature for different potentials. The red dashed line indicates the location of the experimentally measured melting point at about 2128 K.

Figure 7a presents the predicted temperature-dependent thermal expansion coefficients of Zr using different potentials, compared with experimental data. The results show that traditional empirical potentials, such as Zr_M07, exhibit an obvious abnormal contraction trend in the low-to-mid temperature range, and the thermal expansion coefficient even drops below 10, which is a serious deviation from the experiment, indicating their limitations in dealing with thermal perturbations and lattice stability. Zr_A95, Zr_Zhou04, and Zr_Zhou22 generally overestimate the thermal expansion coefficient in the low-to-mid temperature range. In addition, all empirical potentials exhibit varying degrees of numerical divergence at high temperatures (>1200 K), where the thermal expansion coefficient increases abnormally rapidly with temperature, deviating from the experimental curve and showing a nonlinear amplification trend. This phenomenon indicates that empirical potentials struggle to accurately describe the nonlinear enhancement of interatomic interactions and thermal softening effects under strong thermal perturbations at high temperatures, resulting in a significant decline in their thermodynamic prediction capability. In contrast, DP demonstrates excellent agreement with experimental data across the entire temperature range. Between 300 and 800 K, the thermal expansion coefficient predicted by DP remains close to 6 × 10^−6^ K^−1^, showing minimal deviation from experimental values and successfully capturing the subtle expansion behavior under low-energy thermal perturbations. At higher temperatures (>1100 K), DP continues to exhibit stable and physically reasonable thermal expansion behavior, with the coefficient gradually increasing in a smooth nonlinear fashion without any divergence or abnormal fluctuations. This performance reflects high accuracy and fidelity of the DP model in describing lattice dynamics across a wide temperature range.

Zr exhibits excellent thermal stability and high-temperature resistance, with a melting point of approximately 2128 K. Additionally, there exists a two-phase coexistence region near its melting point, involving significant atomic rearrangement, liquid-phase diffusion behavior, and energy barriers. This not only provides a means to validate the accuracy of interatomic potentials under extreme conditions but also tests their generalization capability in complex multiphase systems with intense structural changes. During the melting process, Zr first undergoes a solid–solid phase transition from HCP-Zr to BCC-Zr at around 1408 K, and then melts into a disordered liquid metal structure at approximately 2128 K. As shown in Figure 7b, all potentials predict a general trend of increasing volume with temperature, reflecting the typical thermal expansion behavior of solids and aligning with fundamental physical principles. However, significant differences emerge in the high-temperature region, revealing variations in their ability to capture melting behavior. Zr_A95, Zr_Zhou04, and Zr_Zhou22 potentials show certain volume discontinuities near the experimental melting point, but the changes are relatively moderate, suggesting that the predicted melting points are slightly underestimated or that the phase transitions are not sufficiently pronounced. In contrast, Zr_M07 potential exhibits a more abrupt volume increase, but the transition occurs around 1775 K, which is well below the experimental melting point. In comparison, the melting point predicted by the DP appears close to the experimental value and is accompanied by a distinct volume expansion, consistent with the physical characteristics of density reduction during melting. This indicates that DP demonstrates superior sensitivity and physical fidelity in capturing solid–liquid phase transitions, and it effectively covers both solid and liquid structural features, showcasing strong generalization capability across phase boundaries.

#### 3.3.2. Elastic Constant

Zr has a HCP structure at room temperature and pressure, and its elastic properties are characterized by a pronounced anisotropy. For the HCP structure, there are five independent elastic constants: C_11_, C_12_, C_13_, C_33_, and C_44_. Among them, C_11_ and C_33_ represent the longitudinal stiffness along the *a*-axis and *c*-axis, respectively. C_12_ and C_13_ describe the stress–strain coupling between different crystallographic directions, and C_44_ is associated with shear deformation. The remaining commonly used elastic constants, C_22_, C_23_, C_55_, and C_66_, can be derived from symmetry relations as C_22_ = C_11_, C_23_ = C_13_, C_55_ = C_44_, and the shear modulus C_66_ = (C_11_ − C_12_)/2. Based on the above elastic constants, important mechanical parameters reflecting the overall mechanical behavior, such as bulk modulus (B_V_) and shear modulus (G_V_), can be further derived. The expression for the bulk modulus is

B_V_ = (2C_11_ + 2C_12_ + C_33_ + 4C_13_)/9 (3)

The shear modulus is calculated as

G_V_ = (C_44_ + C_55_ + C_66_)/3 (4)

Table 2 compares the performance of DP with four empirical potentials in predicting the HCP-Zr elastic constants, using the results of DFT calculations as a reference standard. The table lists nine elastic constants C_11_, C_12_, C_13_, C_22_, C_23_, C_33_, C_44_, C_55_, C_66_, as well as bulk modulus B_V_ and shear modulus G_V_. From the results, it can be seen that the DP predictions of all the elastic constants have very little deviation from the DFT results, and the results of C_11_ and C_22_ are completely in agreement with the DFT results. This shows that DP has high accuracy and consistency in describing the elastic response of the crystal in the main-axis direction, as well as the behavior of the inter-axial coupling. In contrast, the elastic constants of other traditional potentials have systematic deviations in multiple directions. For example, Zr_M07 and Zr_Zhou22 significantly overestimate the longitudinal modulus, while the Zr_Zhou04 potential generally underestimates the stiffness parameters, resulting in a low bulk modulus of 87 GPa and a low shear modulus of 28 GPa, respectively. The shear modulus of Zr_A95, although it is close to that of the DFT in some of the parameters, has a significant overestimation of the shear modulus C_44_ and C_55_. In summary, DP performs closest to the first principles results in terms of anisotropic elastic behavior and overall mechanical property prediction, which proves its high reliability in the simulation of structural mechanical properties.

#### 3.3.3. Tensile Properties

While the elastic constants characterize the local response under small strains, the tensile process involves a larger range of structural rearrangements, dislocation activities, twin formation, and even phase transition behaviors, which requires a higher capability of describing the potential energy surface of the potential under large deformation conditions. Therefore, this study performed uniaxial tensile simulations on the HCP-Zr with 223,200 atoms using five interatomic potentials (Zr_A95, Zr_M07, Zr_Zhou04, Zr_Zhou22, and Zr_DP).

**Figure 8:**
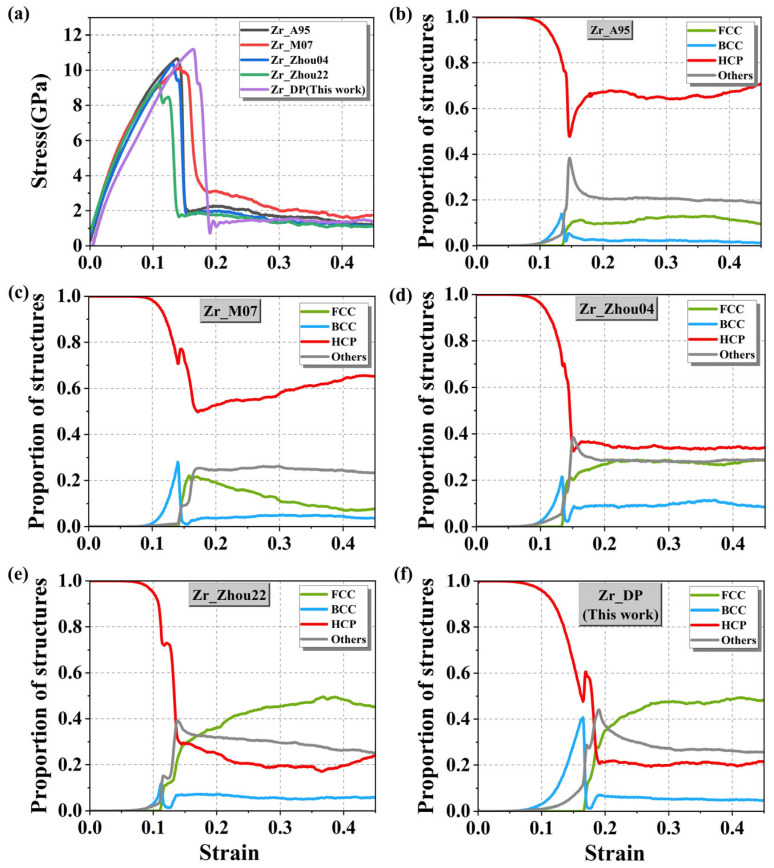
(**a**) Stress–strain curves of HCP-Zr under uniaxial tension along the *x*-axis simulated using different interatomic potentials, Volume fraction evolution of different crystal phases during the tensile simulations based on (**b**) Zr_A95; (**c**) Zr_M07; (**d**) Zr_Zhou04; (**e**) Zr_Zhou22 and (**f**) Zr_DP (This work).

First, the initial HCP crystal structure was subjected to 10,000 steps of thermodynamic relaxation under the isothermal-isobaric (NPT) ensemble to stabilize the system at the target temperature of 300 K and zero pressure. Then, uniaxial tension was applied along the *x*-axis at a constant engineering strain rate of 10^−3^ ps^−1^ for 500,000 steps. The resulting stress–strain curves are shown in Figure 8a. In the elastic regime, all potentials exhibit a clear linear relationship between stress and strain, indicating that they can accurately capture the stiffness characteristics of the material within the small-strain range. However, as the strain continues to increase, the systems reach their yield limits around ε ≈ 0.05 and subsequently enter the nonlinear plastic deformation stage. The stress–strain curves begin to diverge significantly depending on the potential used, reflecting different abilities of the potentials to model large-strain behavior and structural rearrangements. Figure 8b–f show that when the strain increases to the range of ε ≈ 0.1 to 0.2, all the potentials predict a rapid decrease in the fraction of the HCP phase and a corresponding sharp increase in the fraction of the FCC phase during the tensile process. However, in Figure 8b–d, the FCC phase remains at a low fraction throughout, with the HCP phase still dominating. This indicates that these three potentials overestimate the energy barrier for the HCP-to-FCC phase transition. In contrast, Figure 8e,f show a significant increase in the fraction of the FCC phase, indicating that the Zr_Zhou22 and DP potentials are more effective in capturing the stress-induced phase transition process. Therefore, we quantitatively calculated the phase transition energy barrier value obtained from the DP prediction and found it to be 0.373 eV, which is highly consistent with the DFT calculation value 0.16 eV [57]. In contrast, the phase-transition barriers obtained from empirical potentials are clearly unphysical, including 340.932 eV (Zr_A95), 24.725 eV (Zr_Zhou04), and 341.213 eV (Zr_Zhou22). Additionally, DP (Figure 8f) highlights a distinct evolution trajectory of the BCC phase, which peaks at around 40% near ε ≈ 0.16 and then sharply drops to below 10%. This suggests that the BCC phase exists only transiently during the plastic response of the system and serves as an intermediate structure. Its formation is closely related to local atomic rearrangements driven by stress fields during slip. As the strain continues to increase, the BCC phase rapidly transforms into the more stable FCC phase, thus completing the HCP-FCC transition path. This also supports experimental observations [59], which indicate that the HCP-to-FCC transformation is not a simple and abrupt jump but rather a process involving multiple local slips and stacking reorganizations.

**Figure 9:**
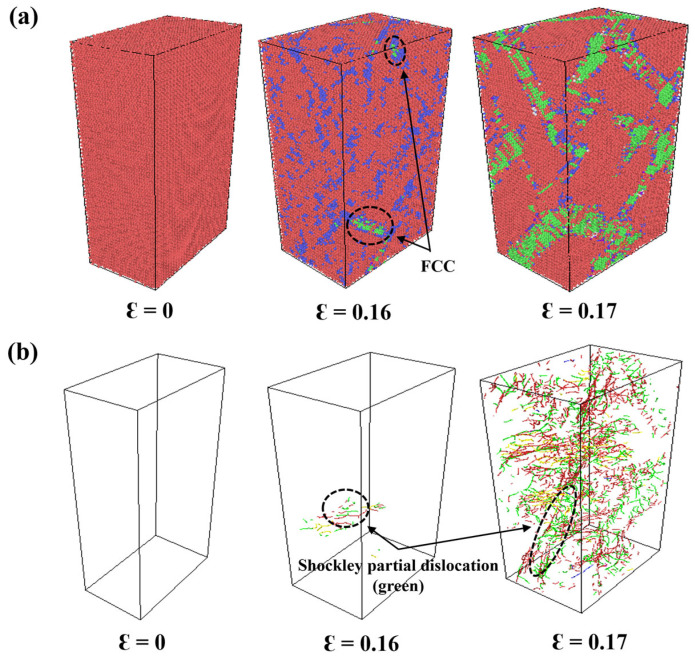
(**a**) Evolution of atomic configurations under different strain stages based on DP simulations. (**b**) Dislocation structure evolution process based on DP simulations based on DXA identification.

To further validate the accuracy of the DP in predicting the mechanical behavior of Zr, this study investigates the dynamic evolution during the tensile deformation of HCP-Zr, revealing the characteristics of the HCP-FCC phase transition. Figure 9a,b show that when the strain reaches approximately ε ≈ 0.16, small-scale lamellar structures (in green) appear within the HCP matrix, accompanied by a high density of dislocations in the surrounding area. This indicates that plastic deformation is localized in this region, inducing a local transformation of the crystal structure. As the tensile strain increases further, the proportion of FCC structures continues to grow, as illustrated in Figure 9b. At ε ≈ 0.17, FCC atoms form pronounced parallel or staggered layered bands, extending along specific crystallographic directions. Dislocation analysis reveals the presence of numerous Shockley partial dislocations within the matrix, gliding along the {0001} basal planes. This dislocation-driven slip mechanism, characterized by high dislocation density and layered stacking glide, directly leads to the transformation of the HCP stacking sequence from ABAB to the ABCABC pattern typical of FCC structures, which is a typical B-type HCP-FCC phase transition mechanism. The DP simulation results show strong agreement with experimental observations reported in the literature [59], demonstrating the excellent predictive accuracy and physical fidelity of DP in describing stress-induced phase transitions in HCP-Zr.

## 4. Conclusions

This work develops a high-accuracy machine learning interatomic potential for Zirconium based on the DP model. The performance of the developed DP was evaluated by comparing it with four representative empirical potentials (Zr_A95, Zr_M07, Zr_Zhou04, and Zr_Zhou22), DFT calculations, and experimental results across seven key physical properties, including lattice constants, surface energy, grain boundary energy, thermal expansion coefficient, melting point, elastic constants, and tensile stress–strain behavior. The results demonstrate that DP exhibits excellent predictive capability in terms of structural properties, with lattice constants highly consistent with DFT results. It also outperforms traditional empirical potentials in accurately capturing the characteristics of non-equilibrium and defect structures, such as surface energy and grain boundary energy, showing superior accuracy and anisotropy resolution. It is worth noting that the predicted surface and grain boundary configurations were not included in the training dataset, highlighting the strong transferability of DP to unseen structures.

Regarding thermodynamic behavior, the predicted melting point is the closest to the experimental value, accurately reproducing the melting behavior of Zr at high temperatures. This indicates the capability of DP to capture complex thermodynamic processes involving large atomic rearrangements, solid–liquid coexistence, and energy barrier transitions. In terms of mechanical properties, DP accurately reproduces the elastic constants tensor of HCP-Zr. Furthermore, in uniaxial tensile simulations, it not only consistently captures the peak stress but also reasonably represents the subsequent stress drop, plastic softening, and stress plateau behavior after yielding. In particular, during the stress-induced HCP-FCC phase transition, DP accurately captures the formation of the FCC phase at the yielding stage, the generation and glide of Shockley partial dislocations, and the transformation of the lattice stacking sequence. These results are highly consistent with experimental observations, demonstrating the remarkable generalization capability of the developed Zr DP in terms of large deformation, local structural rearrangement, and defects evolution.

Although the developed Zr DP model demonstrates excellent consistency and re-liability in predicting various physical properties, certain limitations warrant attention. First, while the computational efficiency of this model far exceeds that of DFT, enabling effective support for medium to large-scale MD simulations, it remains less efficient than traditional empirical potentials. This results in higher computational costs for long-time-scale or ultra-large-system simulations. Second, as machine learning potentials are inherently data-driven models, their extrapolation stability and physical interpretability beyond the training data distribution require improvement, particularly when introducing foreign elements to form alloys or doped systems. Due to the emergence of new chemical environments within the system, the potential energy surface representation of the original model often becomes ineffective. It is necessary to reconstruct the training set and retrain the model to ensure accuracy and robustness in the new chemical space. Future research will focus on enriching datasets by introducing multicomponent alloys or different structures of doped systems. Integrating active learning and uncertainty assessment methods will further enhance the accuracy, robustness, and generalization capabilities of the model.

## Figures and Tables

**Table 1:** Comparison of DP with four empirical potentials, DFT calculations, and experimental data [53] for the prediction of the fundamental physical properties of Zr.

Property	A95	M07	Zhou04	Zhou22	DP	DFT	NNP4 [54]	Experiments
Lattice parameters (Å)								
a	3.22	3.21	3.2	3.22	3.22	3.23	3.231	3.229
c	5.13	5.12	5.10	5.14	5.14	5.15	5.18	5.141
Surface energies (J/m^2^)								
(0001)	1.022	1.360	1.271	1.122	1.495	1.589	—	—
(1010)	1.234	1.532	1.488	1.377	1.877	1.791	—	—
(11$\overset{-}{2}$0)	1.220	1.529	1.480	1.362	1.809	1.786	—	—
(11$\overset{-}{2}$1)	1.266	1.558	1.530	1.415	1.844	1.836	—	—
GB energies (mJ/m^2^)								
(10$\overset{-}{1}$1)	120.93	124.80	92.87	189.47	134.74	150.90	94	—
(10$\overset{-}{1}2$)	198.09	329.23	258.48	285.75	263.34	265.24	270	—
(11$\overset{-}{2}2$)	199.14	282.65	219.12	300.51	281.09	249.64	290	—
Vacancy migration energy barrier (eV)								
basal	1.700	1.180	1.518	1.317	0.757	0.54 [55]	—	—
c	1.603	1.195	1.429	1.250	0.823	0.65 [55]	—	—
Interstitial atom migration energy barrier (eV)								
basal	4.418	1.377	0	3.228	0.632	0.27 [56]	—	—
Phase transition barrier (eV)								
HCP-FCC	340.932	-	24.725	341.213	0.373	0.16 [57]	—	—

**Table 2:** The elastic constants, bulk modulus, and shear modulus of HCP-Zr for different interatomic potentials and DFT calculations.

Properties (GPa)	DFT	DP	A95	M07	Zhou04	Zhou22	NNP4 [54]
C_11_	145	145	159	184	127	178	141.5
C_12_	84	91	79	90	67	92	67.2
C_13_	66	64	65	71	59	77	67.2
C_22_	145	145	159	184	127	178	—
C_23_	66	64	65	71	59	77	—
C_33_	180	179	154	196	158	207	168.7
C_44_	23	19	34	44	27	33	28.9
C_55_	23	19	34	44	27	33	—
C_66_	31	27	40	47	30	43	—
B_V_	115	101	99	114	87	117	—
G_V_	26	22	36	45	28	36	—

## Data Availability

The original contributions presented in this study are included in the article. Further inquiries can be directed to the corresponding authors.

## References

[1] Stoll U., Slavinskaya N. (2023). Corrosion behavior of zirconium alloys in the aqueous environment. Phenomenological aspects. Overview. J. Nucl. Sci. Technol..

[2] Jarugula R., Halodová P., Zimina M., Sundararaman M., Malá M., Klouzal J., Ševeček M., Běláč J., Řeháček R., Linhart S. (2025). Effect of neutron irradiation on microstructural evolution and deformation behavior of Zirconium (Zr-1Nb) alloy. J. Nucl. Mater..

[3] Warwick A.R., Boleininger M., Dudarev S.L. (2021). Microstructural complexity and dimensional changes in heavily irradiated zirconium. Phys. Rev. Mater..

[4] Zhang L. (2021). Understanding the Radiation Resistance Mechanisms of Nanocrystalline Metals from Atomistic Simulation. Metals.

[5] Onimus F., Doriot S., Ambard A., Bourlier F., Verhaeghe B., Le Jolu T., Cappelaere C. (2024). Understanding post-irradiation creep behavior of M5Framatome zirconium alloy. J. Nucl. Mater..

[6] Ding Y., Guo L., Li Y., Cui D., Chang X., Han Q., Deng H., Ran G. (2025). Exploring irradiation-induced HCP to FCC phase transformation in a micro-grained zirconium alloy. J. Nucl. Mater..

[7] Bell S.B., Ridley M.J., Massey C.P., Capps N.A. (2025). Step-loaded creep testing of Zircaloy-4 cladding at higher temperatures in the α-phase. Acta Mater..

[8] Moore B., Topping M., Long F., Daymond M.R. (2025). Stress and temperature dependence of irradiation creep in zircaloy-4 studied using proton irradiation. J. Nucl. Mater..

[9] Jiao Y.-J., Li Z.-Y., Pu Z.-P., Zheng M.-Y., Ren Q.-Y., Cai Z.-B., Wu Y.-W., Qiu S.-Z. (2025). Study on the fretting wear performance of oxide layer and Cr coating on zirconium alloy in high-temperature water. Wear.

[10] Zhao S., Pan R., Zhang Y., Liu H., Wu L., Yang J. (2025). Unveiling the synergistic effects of irradiation-induced defects and the β-Nb phase on the corrosion behavior of Nb-containing Zr-alloys from TEM observation. Mater. Today Commun..

[11] Topping M., Harte A., Ungár T., Race C.P., Dumbill S., Frankel P., Preuss M. (2019). The effect of irradiation temperature on damage structures in proton-irradiated zirconium alloys. J. Nucl. Mater..

[12] Chirkov P.V., Eltsov G.S., Karavaev A.V., Dremov V.V., Mirzoev A.A. (2024). Zirconium phase diagram from ab initio molecular dynamics. Comput. Mater. Sci..

[13] Torres E., Maxwell C. (2023). Effect of irradiation damage on the tensile deformation of α-zirconium systems: A molecular dynamics study. Comput. Mater. Sci..

[14] Khiara N., Onimus F., Dupuy L., Kassem W., Crocombette J.-P., Pardoen T., Raskin J.-P., Bréchet Y. (2020). A novel displacement cascade driven irradiation creep mechanism in α-zirconium: A molecular dynamics study. J. Nucl. Mater..

[15] Zhang L., Zhang Z., Zhang X., Huang X. (2022). Computational simulation of grain boundary segregation of solute atoms in nanocrystalline metals. J. Mater. Res. Technol..

[16] Zhang X., Zhang L., Zhang Z., Huang X. (2023). Effect of solute atoms segregation on Al grain boundary energy and mechanical properties by first-principles study. Mech. Mater..

[17] Zhang X., Zhang L., Wan Y., Shibuta Y., Huang X. (2024). Predicting the grain boundary segregation energy of solute atoms in aluminum by first-principles calculation and machine learning. Mater. Today Commun..

[18] del Rio B.G., Phan B., Ramprasad R. (2023). A deep learning framework to emulate density functional theory. npj Comput. Mater..

[19] Zhang L., Mao W., Liu M., Shibuta Y. (2020). Mechanical response and plastic deformation of coherent twin boundary with perfect and defective structures. Mech. Mater..

[20] Liao Z., Zhang L., Hou X., Huang X. (2025). Grain boundary segregation of solutes and associated plastic deformation mechanisms in nanocrystalline Al–Cu and Al–Mg alloys: A molecular dynamics study. J. Mater. Res. Technol..

[21] Berendsen H.J.C., Deuflhard P., Hermans J., Leimkuhler B., Mark A.E., Reich S., Skeel R.D. (1999). Molecular Dynamics Simulations: The Limits and Beyond. Computational Molecular Dynamics: Challenges, Methods, Ideas.

[22] Xie S.R., Rupp M., Hennig R.G. (2023). Ultra-fast interpretable machine-learning potentials. npj Comput. Mater..

[23] Choudhary K., Congo F.Y., Liang T., Becker C., Hennig R.G., Tavazza F. (2017). Evaluation and comparison of classical interatomic potentials through a user-friendly interactive web-interface. Sci. Data.

[24] Wang G., Wang C., Zhang X., Li Z., Zhou J., Sun Z. (2024). Machine learning interatomic potential: Bridge the gap between small-scale models and realistic device-scale simulations. iScience.

[25] Mendelev M.I., Ackland G.J. (2007). Development of an interatomic potential for the simulation of phase transformations in zirconium. Philos. Mag. Lett..

[26] Nicholls O.G., Frost D.G., Tuli V., Smutna J., Wenman M.R., Burr P.A. (2023). Transferability of Zr-Zr interatomic potentials. J. Nucl. Mater..

[27] Behler J. (2014). Representing potential energy surfaces by high-dimensional neural network potentials. J. Phys. Condens. Matter.

[28] Min H., Wu F., Yang J., Duan X., Wen Y., Xie F., Shan B. (2021). Development of an interatomic potential for Fe-He by neural network. Comput. Mater. Sci..

[29] Konashi K., Kato N., Mori K., Kurosaki K. (2025). Neural network potential for molecular dynamics calculation of UO_2_. J. Nucl. Mater..

[30] Li H., Wu L., Xia C., Huang S., Ni M., Huang C., Xu M., Ruan Z. (2025). A deep neural network potential model for theoretically predicting thermal transport, mechanical properties of multi-layered graphitic carbon nitride with molecular dynamics. Int. Commun. Heat Mass Transf..

[31] Wang H., Zhang L., Han J., E W. (2018). DeePMD-kit: A deep learning package for many-body potential energy representation and molecular dynamics. Comput. Phys. Commun..

[32] Zeng J., Zhang D., Lu D., Mo P., Li Z., Chen Y., Rynik M., Huang L.a., Li Z., Shi S. (2023). DeePMD-kit v2: A software package for deep potential models. J. Chem. Phys..

[33] Meng X.Y., Wang X., Li M.Z., Tan G.M., Jia W.L. (2025). An interpretable DeePMD-kit performance model for emerging supercomputers. CCF Trans. High Perform. Comput..

[34] Wang X., Wang Z., Gao P., Zhang C., Lv J., Wang H., Liu H., Wang Y., Ma Y. (2023). Data-driven prediction of complex crystal structures of dense lithium. Nat. Commun..

[35] Wang H., Li T., Yao Y., Liu X., Zhu W., Chen Z., Li Z., Hu W. (2023). Atomistic modeling of lithium materials from deep learning potential with ab initio accuracy. Chin. J. Chem. Phys..

[36] Xue H.-T., Li J., Chang Z., Yang Y.-H., Tang F.-L., Zhang Y., Ren J.-Q., Lu X.-F., Li J.-C. (2024). Deep-learning potential molecular dynamics simulations of the structural and physical properties of rare-earth metal scandium. Comput. Mater. Sci..

[37] Ding C.-J., Lei Y.-W., Wang X.-Y., Li X.-L., Li X.-Y., Zhang Y.-G., Xu Y.-C., Liu C.-S., Wu X.-B. (2024). A deep learning interatomic potential suitable for simulating radiation damage in bulk tungsten. Tungsten.

[38] Wang H., Zhang Y., Zhang L., Wang H. (2020). Crystal Structure Prediction of Binary Alloys via Deep Potential. Front. Chem..

[39] Chen H.M., Wang Q., Xiao R.L., Wang H.P. (2024). Liquid thermophysical properties of Ag-Si alloy based on deep learning potential. Comput. Mater. Sci..

[40] Zhu C.-s., Dong W.-j., Gao Z.-h., Wang L.-j., Li G.-z. (2024). Deep Potential fitting and mechanical properties study of MgAlSi alloy. Comput. Mater. Sci..

[41] Jiang W., Zhang Y., Zhang L., Wang H. (2021). Accurate Deep Potential model for the Al–Cu–Mg alloy in the full concentration space. Chin. Phys. B.

[42] Momma K., Izumi F. (2011). VESTA 3 for three-dimensional visualization of crystal, volumetric and morphology data. J. Appl. Crystallogr..

[43] Kresse G., Furthmüller J. (1996). Efficient iterative schemes for ab initio total-energy calculations using a plane-wave basis set. Phys. Rev. B.

[44] Perdew J.P., Burke K., Ernzerhof M. (1996). Generalized Gradient Approximation Made Simple. Phys. Rev. Lett..

[45] Monkhorst H.J., Pack J.D. (1976). Special points for Brillouin-zone integrations. Phys. Rev. B.

[46] Chen J.W., Yu C.M., Kao C.C., Pang T.W., Lu C.S. DPGEN: Differentially Private Generative Energy-Guided Network for Natural Image Synthesis. Proceedings of the 2022 IEEE/CVF Conference on Computer Vision and Pattern Recognition (CVPR).

[47] Wang Y., Ding Z., Xiao Y., Kifer D., Zhang D. DPGen: Automated Program Synthesis for Differential Privacy. Proceedings of the 2021 ACM SIGSAC Conference on Computer and Communications Security, Association for Computing Machinery.

[48] Wen T., Zhang L., Wang H., E W., Srolovitz D.J. (2022). Deep potentials for materials science. Mater. Futures.

[49] Zhang L., Lin D.-Y., Wang H., Car R., E W. (2019). Active learning of uniformly accurate interatomic potentials for materials simulation. Phys. Rev. Mater..

[50] Ackland G., Wooding S., Bacon D. (1995). Defect, surface and displacement-threshold properties of α-zirconium simulated with a many-body potential. Philos. Mag. A.

[51] Zhou X.W., Johnson R.A., Wadley H.N.G. (2004). Misfit-energy-increasing dislocations in vapor-deposited CoFe/NiFe multilayers. Phys. Rev. B.

[52] Zhou M., Fu B., Hou Q., Wu L., Pan R. (2022). Determining the diffusion behavior of point defects in zirconium by a multiscale modelling approach. J. Nucl. Mater..

[53] Goldak J., Lloyd L.T., Barrett C.S. (1966). Lattice Parameters, Thermal Expansions, and Grüneisen Coefficients of Zirconium, 4.2 to 1130°K. Phys. Rev..

[54] Liyanage M., Reith D., Eyert V., Curtin W.A. (2022). Machine learning for metallurgy V: A neural-network potential for zirconium. Phys. Rev. Mater..

[55] Varvenne C., Mackain O., Clouet E. (2014). Vacancy clustering in zirconium: An atomic-scale study. Acta Mater..

[56] Shi T., Liu W., Zhang C., Lyu S., Sun Z., Peng Q., Li Y., Meng F., Tang C., Lu C. (2024). An investigation of self-interstitial diffusion in α-zirconium by an on-the-fly machine learning force field. AIP Adv..

[57] Njifon I.C., Torres E. (2021). A first principles investigation of the hydrogen-strain synergy on the formation and phase transition of hydrides in zirconium. Acta Mater..

[58] Petukhov V. (2003). Thermal expansion of zirconium in the solid phase. High Temp.-High Press..

[59] Shen Z., Liu C., Liu J., Wu J., Liu Y., Liu N., Zhang Y. (2025). HCP-FCC phase transition behavior of zirconium under straining—An in-situ analysis. J. Alloys Compd..

